# A lightweight Yunnan Xiaomila detection and pose estimation based on improved YOLOv8

**DOI:** 10.3389/fpls.2024.1421381

**Published:** 2024-06-05

**Authors:** Fenghua Wang, Yuan Tang, Zaipeng Gong, Jin Jiang, Yu Chen, Qiang Xu, Peng Hu, Hailong Zhu

**Affiliations:** ^1^Faculty of Modern Agricultural Engineering, Kunming University of Science and Technology, Kunming, Yunnan, China; ^2^Engineering Training Center, Kunming University of Science and Technology, Kunming, Yunnan, China

**Keywords:** improved YOLOv8, Xiaomila fruits, target detection, lightweight, pose estimation

## Abstract

**Introduction:**

Yunnan Xiaomila is a pepper variety whose flowers and fruits become mature at the same time and multiple times a year. The distinction between the fruits and the background is low and the background is complex. The targets are small and difficult to identify.

**Methods:**

This paper aims at the problem of target detection of Yunnan Xiaomila under complex background environment, in order to reduce the impact caused by the small color gradient changes between xiaomila and background and the unclear feature information, an improved PAE-YOLO model is proposed, which combines the EMA attention mechanism and DCNv3 deformable convolution is integrated into the YOLOv8 model, which improves the model’s feature extraction capability and inference speed for Xiaomila in complex environments, and achieves a lightweight model. First, the EMA attention mechanism is combined with the C2f module in the YOLOv8 network. The C2f module can well extract local features from the input image, and the EMA attention mechanism can control the global relationship. The two complement each other, thereby enhancing the model’s expression ability; Meanwhile, in the backbone network and head network, the DCNv3 convolution module is introduced, which can adaptively adjust the sampling position according to the input feature map, contributing to stronger feature capture capabilities for targets of different scales and a lightweight network. It also uses a depth camera to estimate the posture of Xiaomila, while analyzing and optimizing different occlusion situations. The effectiveness of the proposed method was verified through ablation experiments, model comparison experiments and attitude estimation experiments.

**Results:**

The experimental results indicated that the model obtained an average mean accuracy (mAP) of 88.8%, which was 1.3% higher than that of the original model. Its F1 score reached 83.2, and the GFLOPs and model sizes were 7.6G and 5.7MB respectively. The F1 score ranked the best among several networks, with the model weight and gigabit floating-point operations per second (GFLOPs) being the smallest, which are 6.2% and 8.1% lower than the original model. The loss value was the lowest during training, and the convergence speed was the fastest. Meanwhile, the attitude estimation results of 102 targets showed that the orientation was correctly estimated exceed 85% of the cases, and the average error angle was 15.91°. In the occlusion condition, 86.3% of the attitude estimation error angles were less than 40°, and the average error angle was 23.19°.

**Discussion:**

The results show that the improved detection model can accurately identify Xiaomila targets fruits, has higher model accuracy, less computational complexity, and can better estimate the target posture.

## 1 Introduction

Pepper is one of the three major vegetable crops in the world. Its fruit has rich polyphenols, flavonoids, vitamin C, and other natural active ingredients, with high food value, economic value, and health care value (Zhang, 2023). Currently, pepper-picking equipment mainly consists of various forms of harvesters, such as rod and comb harvesters, unfolding double helix harvesters, drum finger harvesters, and strip comb harvesters (Fan et al., 2023). Xiaomila is a smaller, lighter, crispy, and tender variety of pepper, and its flowers and fruits have the same characteristics. Traditional picking equipment is not only prone to damaging Xiaomila fruits but also cannot adapt to the characteristics of Xiaomila flowers and fruits that are contemporaneous.

**Figure 1:**
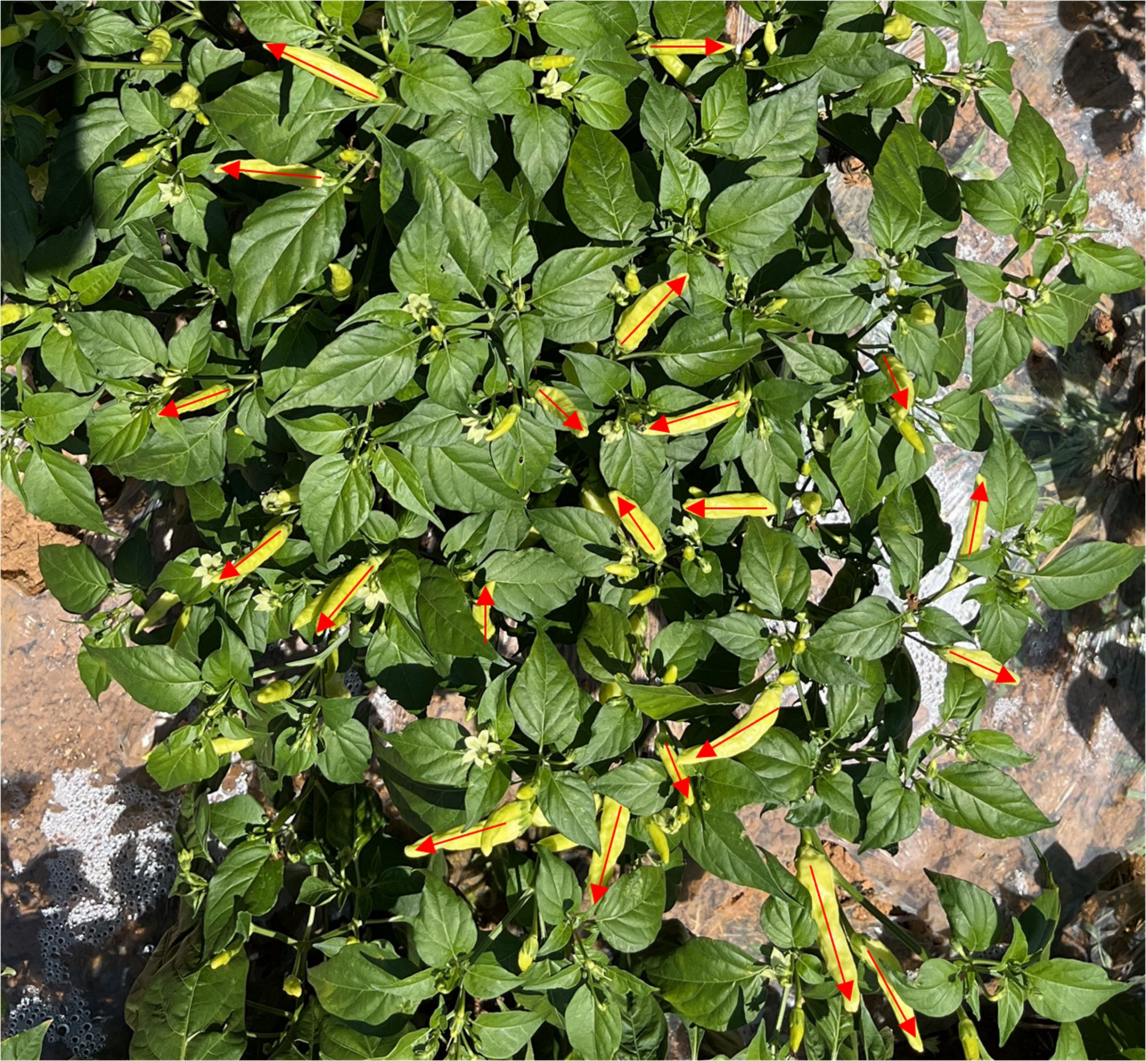
Xiaomila grows in different directions in the natural farmland environment.

In recent years, picking robots have gradually become popular (Ye et al., 2023; Wang et al., 2023a; Tang et al., 2024), different from traditional mechanical picking equipment, picking robots have the capability of non-one-time picking and can reduce uncontrollable damage caused by traditional mechanical equipment. This enables the picking robot to adapt well to the characteristics of Xiaomila flowers and fruits that are contemporaneous and easily damaged. The spatial attitude estimation of Xiaomila objects is the to accurate and collision-free picking, and Xiaomila grows in different directions in the natural farmland environment, as illustrated by the arrows in **Figure 1**.

Attitude estimation is to infer the three-dimensional translation and rotation information of the target in the camera coordinate system from images or videos (Guo et al., 2023). Traditional attitude estimation methods have low applicability in weak texture target detection and real-time detection, while deep learning methods learn feature information in input images through deep neural networks and have high robustness in real-time applications (Lin et al., 2022a). Therefore, current research on target attitude estimation during picking mainly focuses on deep learning methods.

Methods based on RGB-D images generally collect image data containing target depth information through a depth sensor and extract corresponding features for posture regression. Luo et al. obtained the grape cluster image mask and point cloud information using a depth camera, constructed a region of interest based on the mapping relationship between the two, and utilized the LOWESS algorithm and geometric method to fit the pedicel surface and estimate the posture of the pedicel. This estimation method is highly sensitive to point cloud data (Luo et al., 2022). Eizentals et al. obtained green pepper surface point information through a laser rangefinder and obtained the attitude information of the green pepper fruit in space through model fitting, but the accuracy and success rate were not high (Eizentals and Oka, 2016). Yin et al. obtained the grape mask by using the Mask Region Convolutional Neural Network (Mask R-CNN); meanwhile, they combined the RANSAC algorithm to fit the point cloud into a cylindrical model, estimated the grape posture with its axis, and estimated the posture of each bunch of grapes. The approach took about 1.7s to complete the task (Yin et al., 2021). Zhang et al. proposed a tomato bunch attitude detection method for continuous tomato harvesting operations. The method consists of *a priori* model, cascade network, and three-dimensional reconstruction. It fully exploits the advantages of convolutional neural networks while avoiding complex point cloud calculations, but it cannot make correct predictions for fruits with heavy occlusion (Zhang et al., 2022). Lin et al. used RGB-D sensors to obtain binary images of guava and branches through a fully convolutional network, adopted Euclidean clustering to separate different groups of point clouds, and used the guava center and nearest branch information for attitude estimation. However, the success rate and accuracy still need to be improved (Lin et al., 2019). Wang et al. designed a geometric perception network that uses point cloud information and RGB images to detect, segment, and grasp targets. It can better perceive targets, but changes in distance have a greater impact on the estimation accuracy (Wang et al., 2022). Li et al. calculated the local plane normal of each point in the point cloud, scored each candidate plane, took the lowest-scoring plane as the symmetry plane of the point cloud, and calculated the symmetry axis based on this plane to realize attitude estimation of bell peppers. However, the estimation effect is not good for occluded bell peppers (Li et al., 2018).

The input data of the method based on RGB images does not contain depth information, and the features of the image are directly extracted for analysis. Sun et al. constructed a multi-task learning model that locates the position of the citrus navel point and predicts the rotation vector of the citrus by performing RGB image analysis of citrus. However, for citrus whose navel point is invisible, the model needs to be further improved (Sun et al., 2023). Zhang et al. used 3D detection results to regress the 2D key point coordinates of objects in the image. By using the perspective n-point algorithm to estimate the pose of an object, this method enhances the accuracy and efficiency of pose estimation (Zhang et al., 2019). Kim et al. developed a deep learning network for determining robot cutting poses during harvesting, which can perform ripeness classification and pose estimation of fruits and lateral stems. The study results indicate that this method performs well in detecting tomatoes in a smart farm environment. However, the detection effect in complex farmland environments has not been verified (Kim et al., 2022). Based on the growth characteristics of grapes, Wu et al. combined human pose estimation, key point detection models, and target detection algorithms to identify grape clusters and estimate poses. However, this method is not effective for complex image processing (Wu et al., 2023a). Lin et al. analyzed a single RGB image based on key points and estimated the pose of the object by regressing the size of the boundary cuboid, but the network was not sufficiently lightweight (Lin et al., 2022b).

To sum up, the method of using RGB-D images or point cloud data to estimate the pose of a target requires a large amount of calculation and is not suitable for transplantation to mobile devices. Additionally, objects to be identified in farmland are basically occluded. The above methods are usually combined with the stems of the identified objects to realize pose estimation. However, the diameter of Xiaomila stems is very small (1–3 mm), and the background is complex. Traditional stereo cameras and depth sensors such as lidar have been proven to be unable to provide reliable depth information (Coll-Ribes et al., 2023). To solve these problems, this study mainly makes the following contributions:

1) We propose a lightweight, multiscale detection model, called PAE-YOLO, for Xiaomila target detection in complex farmland environments. The EMA attention mechanism can effectively enhance the feature extraction capability of the model, while DCNv3 can significantly reduce the computational complexity of the model and improve the portability of the model.

2) We used a depth camera to detect pepper skins and caps to determine the posture of Xiaomi spicy. We also analyze and optimize Xiaomi target detection and posture determination under different occlusion situations.

3) We determined the effectiveness of the improved model through ablation experiments and comparison experiments, and determined the effectiveness of attitude detection through attitude estimation experiments. Among several classic detection models, our proposed model has higher accuracy, the smallest model size, and the lowest computational effort than several classical models.

## 2 Materials and methods

### 2.1 Image acquisition

This study takes Xiaomila fruits in the green and mature stage of farmland as the research object. All images used in the experiment were taken in 2023 at a Xiaomila plantation in Qiubei County, Wenshan City, Yunnan Province, China. The Intel realsense D435i device was utilized to collect RGB images. During the image collection process, the camera was placed about 15–30 cm away from the Xiaomila plants and photographed directly above the Xiaomila plants. The image resolution was 1920×1080 pixels, and a total of 1060 images were collected.

### 2.2 Dataset construction and annotation

In the natural farmland environment, Xiaomila fruits have a similar color to pepper leaves, with small individuals and complex backgrounds. Considering the difference in images obtained under actual changing lighting and occlusion conditions, the original images are collected at different times, under varying lighting, and with diverse occlusion levels. However, these images typically cannot encompass all real-world conditions. Furthermore, they differ somewhat from actual Xiaomila images. Hence, collected RGB images underwent expansion through random rotation, brightness adjustment, and noise addition to harmonize and mitigate these disparities. In the real environment, the pepper’s orientation varies, and random rotation and flipping primarily serve to diversify its orientation, enhancing the model’s generalization ability. Random clipping accounts for the impact of various occlusion scenarios, ensuring data diversity. Noise addition and brightness adjustment aim to mitigate factors such as brightness deviations among different sensors (Akbar et al., 2022; Bosquet et al., 2023).

Of course, there will still be some differences between the enhanced dataset and the actual changing lighting and occlusion conditions. To minimize such differences, more factors from real scenes can be incorporated when collecting data, such as weather changes, varying occlusion, etc. Additionally, ensuring a similar distribution between training and test data reflects the actual scene more accurately.

**Figure 2:**
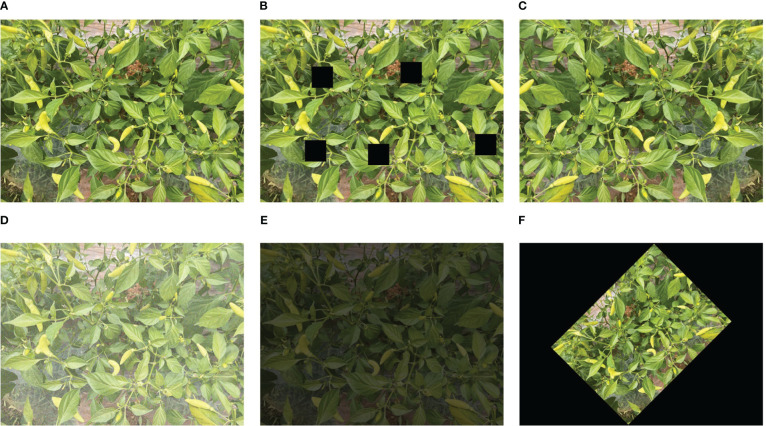
Image expansion effect. **(A)** Original image, **(B)** random cropping, **(C)** flipping, **(D)** noise adding, **(E)** brightness adjustment, **(F)** random rotation.

The expansion effect is demonstrated in **Figure 2**. The final target detection data set consists of 2500 images, of which 1750 are used as a training set and the remaining 750 are used as a verification set. The labeling tool was used to label Xiaomi fruits and convert the labeled xml file into the txt file required by the model.

### 2.3 YOLOv8 network structure

**Figure 3:**
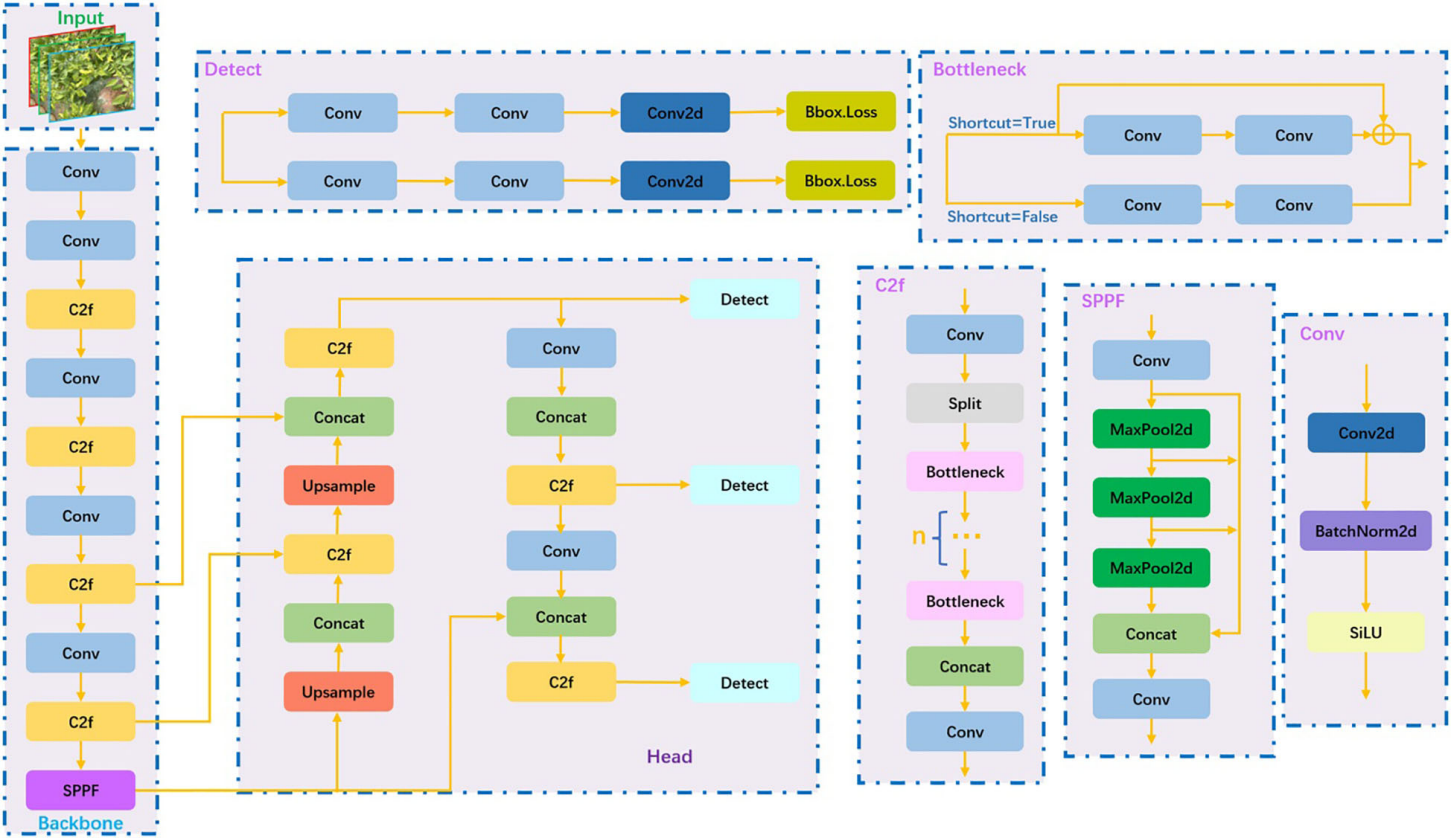
YOLOv8 network architecture.

The YOLO series algorithm is an efficient method with limited computational parameters, making it a key research focus in target detection (Wang et al., 2023b). Wu et al. proposed a segmentation and counting algorithm for banana bunches based on YOLOv5-Banana (Wu et al., 2023b). Song et al. introduced the YOLOv7-ECA model, which offers fast detection speed, specifically designed for the similar color and small size of young apple leaves (Song et al., 2023). Yao et al. presented the SCR-YOLO model for detecting the germination rate of wild rice (Yao et al., 2024). Ranjan et al. utilized the YOLOv8 network to detect and adjust green apples in orchards (Sapkota et al., 2024). YOLOv8 is the latest version of the YOLO series network. According to the scaling coefficient, the network is divided into five scales: n/s/m/l/x. The main updates of the YOLOv8 network lie in the C3 module, head network, and loss function. Specifically, the C3 module is replaced by the C2f module, which improves the backbone network’s ability to fuse the detailed information and semantic information of feature maps at different scales. The original coupling head is replaced with a decoupling head, and the regression branch and prediction branch are separated, leading to better recognition results. Regarding the loss function, YOLOv8 adopts the task-aligned allocator positive sample distribution strategy to optimize the calculation process of the loss function. **Figure 3** shows the overall structure of the YOLOv8 network.

### 2.4 YOLOv8 model improvement strategy

Though YOLOv8 has strong capabilities in target detection, it still has limitations in the detection of Xiaomila fruits. Compared with other crop fruits, Xiaomila fruits exhibit irregular distribution, there is little change in the color gradient between the fruit area and the background, and it is more susceptible to interference from background information. Considering the above limitations, this study improves YOLOv8 in two aspects: attention mechanism and convolutional neural network.

**Figure 4:**
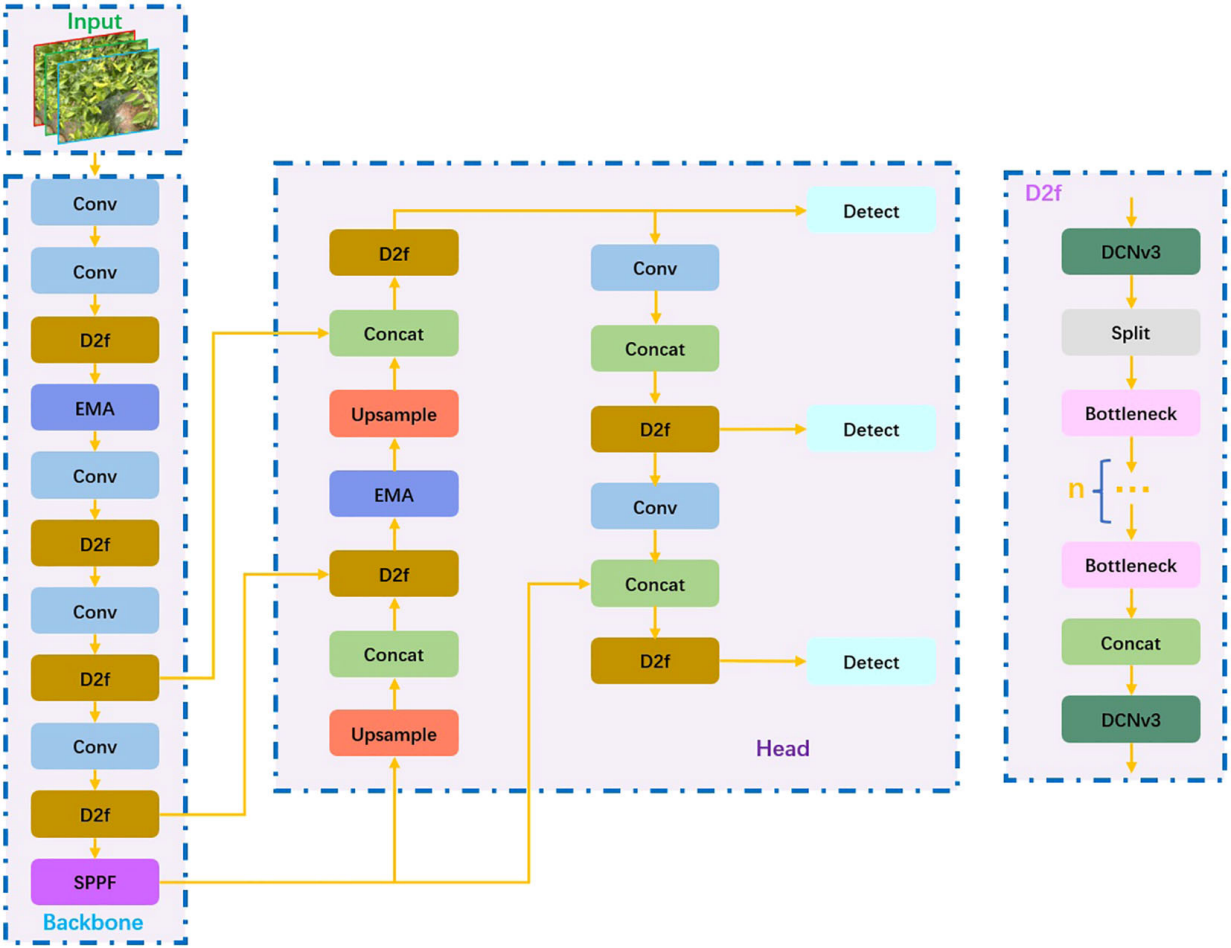
The architecture of the PAE-YOLO network.

First, the EMA attention mechanism is combined with the C2f module in the YOLOv8 network. The C2f module can well extract local features from the input image, and the EMA attention mechanism can control the global relationship. The two complement each other, thereby enhancing the model’s expression ability; Meanwhile, in the backbone network and head network, the DCNv3 convolution module is introduced, which can adaptively adjust the sampling position according to the input feature map, contributing to stronger feature capture capabilities for targets of different scales and a lightweight network. The test results suggest that the improved model has better performance in identifying Xiaomila fruits. Since this model is established based on YOLOv8, the improved model is called PAE-YOLO. **Figure 4** demonstrates the entire network framework of PAE-YOLO.

#### 2.4.1 EMA attention mechanism

The attention mechanism is employed to help the model distinguish important channels and enhance the feature information in the channels, thereby improving the model’s perception and generalization ability of feature information. Traditional attention mechanisms usually produce clear feature information by reducing channel dimensions. However, the reduction of channel dimensions may result in partial information loss and increased errors.

**Figure 5:**
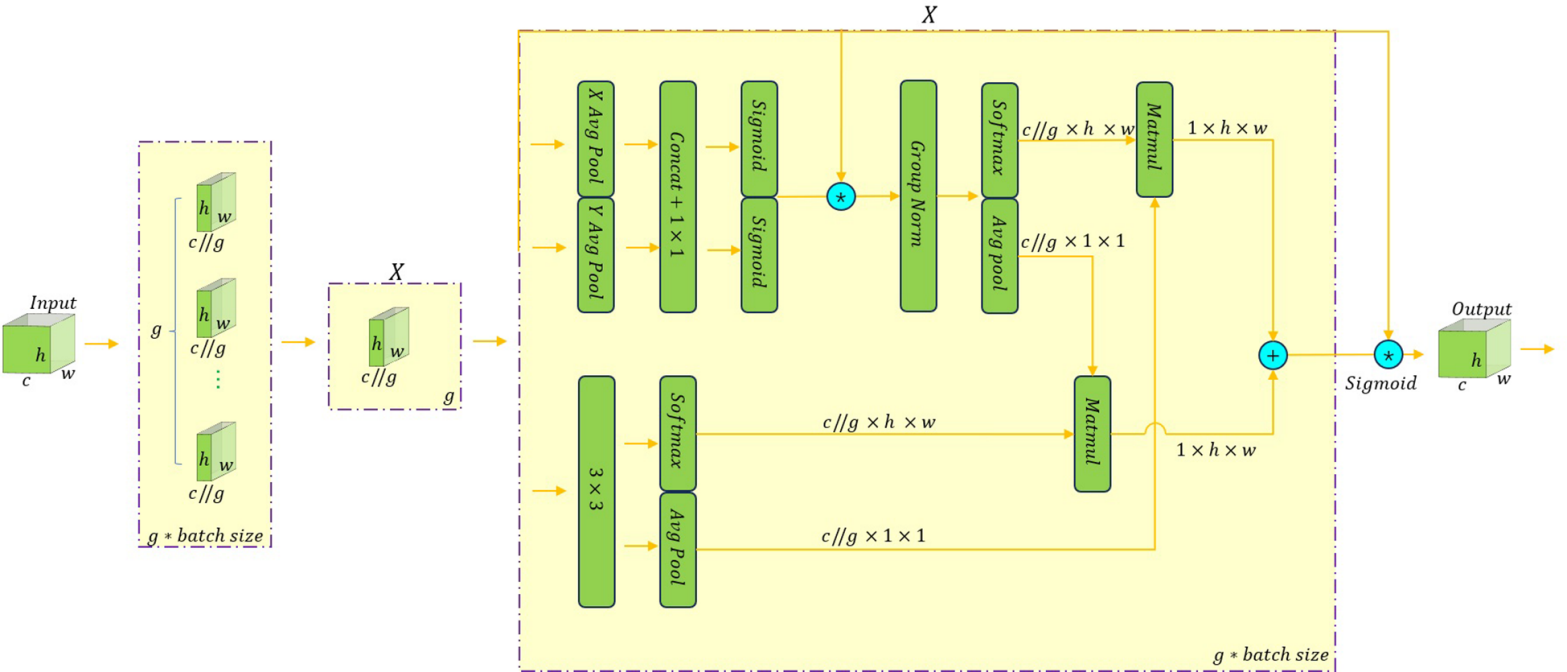
The structure of the EMA attention mechanism.

EMA is a multiscale attention mechanism for calculating attention weights (Ouyang et al., 2023). This mechanism introduces the concept of exponential moving average, which divides each channel of the input image into groups containing multiple sub-features. In the process, the EMA attention mechanism only requires one learning accumulation factor, and the number of added parameters is small, which can guarantee that the spatial semantic features are evenly distributed in each feature group without changing the channel dimension. The specific structure of the attention mechanism is shown in **Figure 5**.

#### 2.4.2 Deformable convolutional network DCNv3

Deformable convolution is a non-fixed sampling convolution network with stronger generalization ability and feature capture ability than ordinary convolution networks. DCNv3 (Wang et al., 2023c) introduces the concept of convolution separation to divide the original convolution weight into two parts: the depth direction and the point direction. The point direction part is taken as the shared projection weight between sampling points to improve the overall efficiency of the model. Meanwhile, DCNv3 divides the process of spatial aggregation into multiple groups with independent sampling offsets and modulation scales. All modulation scalars between sampling points are normalized through softmax, and their sum is constrained to 1, thereby enhancing the training stability of the model. The specific expression is given in Formula 1.


(1) $$\;y\left(p_{0}\right)=\sum_{f=1}^{F}\sum_{h=1}^{H}w_{f}m_{fh}X_{f}\left(p_{0}+p_{h}+\Delta p_{fh}\right)$$

where, F denotes the total number of aggregated groups, H represents the number of dimensions, $w_{f}$ represents the position−independent projection weight of the current group, $m_{fh}$ represents the h sampling points in the f group, $X_{f}$ denotes a slice of the input feature map, $p_{0}$ denotes the current pixel, $p_{h}$ represents the grid sampling position of the current group, and $\text{Δ}p_{fh}$ stands for the offset corresponding to $p_{h}$ .

**Figure 6:**
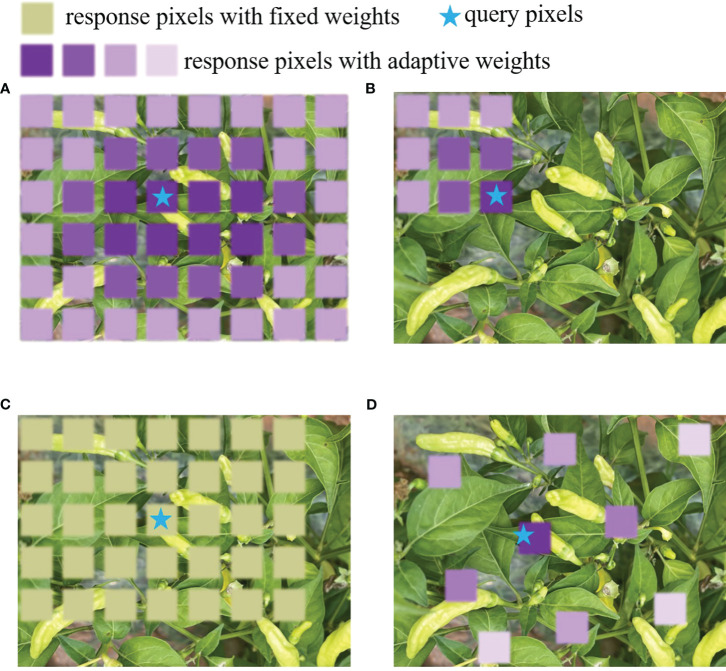
The schematic diagram of different core operators. **(A)** global attention operator, **(B)** local attention operator, **(C)** large kernel operator, **(D)** dynamic sparse kernel operator.

**Figure 6** compares different core operators. (a) shows the global attention operator, which has high computational complexity and memory cost. (b) shows a local attention operator. Although the calculation amount is reduced, it cannot handle long-distance dependencies. (c) shows a large kernel operator, but it cannot adapt to spatial aggregation. (d) shows the dynamic sparse kernel operator used in DCNv3 deformable convolution. It has low computational cost and memory costs, has the capability to handle long-distance dependencies, and can adapt to spatial aggregation.

### 2.5 Posture estimation for Xiaomila fruits

In the natural farmland environment, affected by leaves, branches, and other fruits, the attitude of Xiaomila fruits has little correlation with the fruit itself. Coupled with complex background factors, it is difficult to directly estimate the posture of Xiaomila fruits. This paper adopts the idea of mapping and uses the detection network to identify all the peppers in the image and takes the single pepper image in the recognition frame as the region of interest (RoI). Then, the data of the RoI is passed to the segmentation network, which segments the area target and outputs a binary mask. Next, based on the pixel information of the segmented individual Xiaomila fruits, two-dimensional pose estimation is performed on the Xiaomila fruits, and the pose estimation effect is mapped back to the original image. Finally, combined with the depth information, the spatial posture of Xiaomila fruits is obtained.

#### 2.5.1 Xiaomila 2D fitting

Xiaomila fruits are very light. Unlike heavier crops such as grapefruit and apples, the fruit stems are generally facing downward (Kang et al., 2020; Zeng et al., 2021). Meanwhile, the fruit stems of Xiaomila are very thin and subject to greater interference. These factors make it difficult to directly identify and fit the fruit stems like tomatoes, grapes, lychees, etc (Zhong et al., 2021; Li et al., 2023; Zhang et al., 2023).

**Figure 7:**
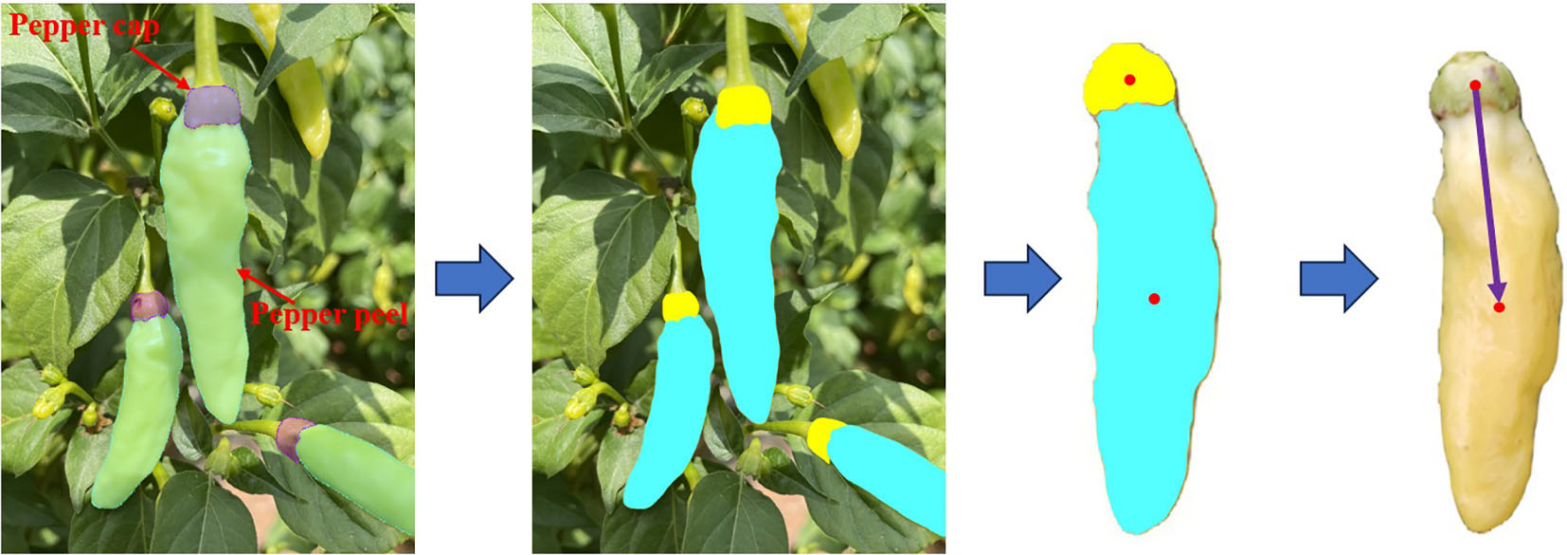
Posture estimation of unoccluded Xiaomila fruits.

There is an obvious gradient change in the color of the pepper peel and the color of the pepper cap. Based on this characteristic, this paper segments the pepper peel and the pepper cap respectively, calculates the moments of the masks of these two parts, and then takes the two-dimensional vector composed of these two moment points as the two-dimensional image posture of Xiaomila fruits, as shown in **Figure 7**.

In the farmland environment, part of the pepper caps are blocked, and the moment points of the pepper caps cannot be successfully obtained. Considering that the Xiaomila fruit is strip-shaped, this paper employs the least squares method (de Jong, 1993) to optimally fit the mask data of the Xiaomila fruit. The relevant parameters and definitions of Xiaomila fruit fitting are given in Formulas 2–4:


(2) $$y=\hat{k}x+\hat{b}$$

(3) $$\hat{k}=\;\frac{\sum_{i=1}^{n}\left(x_{i}-\bar{x}\right)\left(y_{i}-\bar{y}\right)}{\sum_{i=1}^{n}{\left(x_{i}-\bar{x}\right)}^{2}}$$

(4) $$\hat{b}=\bar{y}-\hat{k}\bar{x}$$

where, $x_{i}$ is the x-direction coordinate of the mask outline pixel in the Xiaomila image coordinate system, $y_{i}$ is the y-direction coordinate of the mask outline pixel, n denotes the number of mask outline pixel points, $\bar{x}$ is the x coordinate of all outline pixels. $\bar{y}$ represents the mean of all y-coordinates of the contour pixel. $\hat{k}$ denotes the slope of the mask profile fitting straight line, and $\hat{b}$ is the intercept of the straight line.

**Figure 8:**
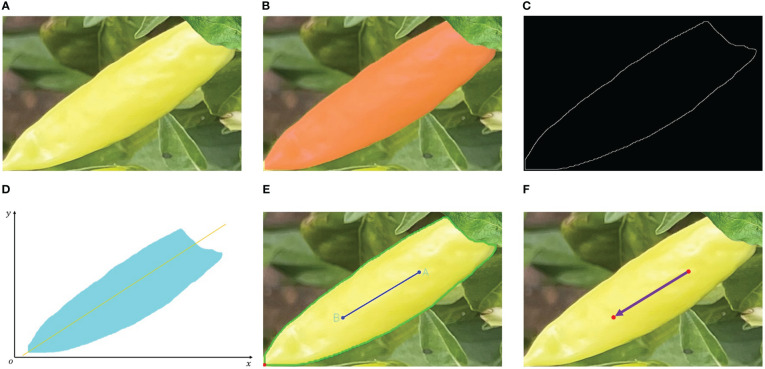
Posture fitting of occluded pepper caps. **(A)** original Xiaomila image, **(B)** mask image of Xiaomila, **(C)** extracted mask contour binary image, **(D)** schematic diagram of contour fitting, **(E)** fitting effect, **(F)** posture effect.

The final fitting effect is illustrated in **Figure 8**. Specifically, (a) shows the original Xiaomila image; (b) shows the mask image of Xiaomila; (c) shows the extracted mask contour binary image; (d) shows a schematic diagram of contour fitting; (e) shows a fitting effect diagram, where the green line represents the Xiaomila contour line, the blue line AB represents the fitting straight line, and the red dot indicates the estimated tip of Xiaomila; (f) shows the posture effect.

Finally, by comparing the sum of the Euclidean distances between the two end points of the contour and other points on the contour to determine which end is the tip, two-dimensional pose estimation of Xiaomila fruits with the pepper cap occluded is realized.

#### 2.5.2 Estimating space posture for Xiaomila fruits

**Figure 9:**
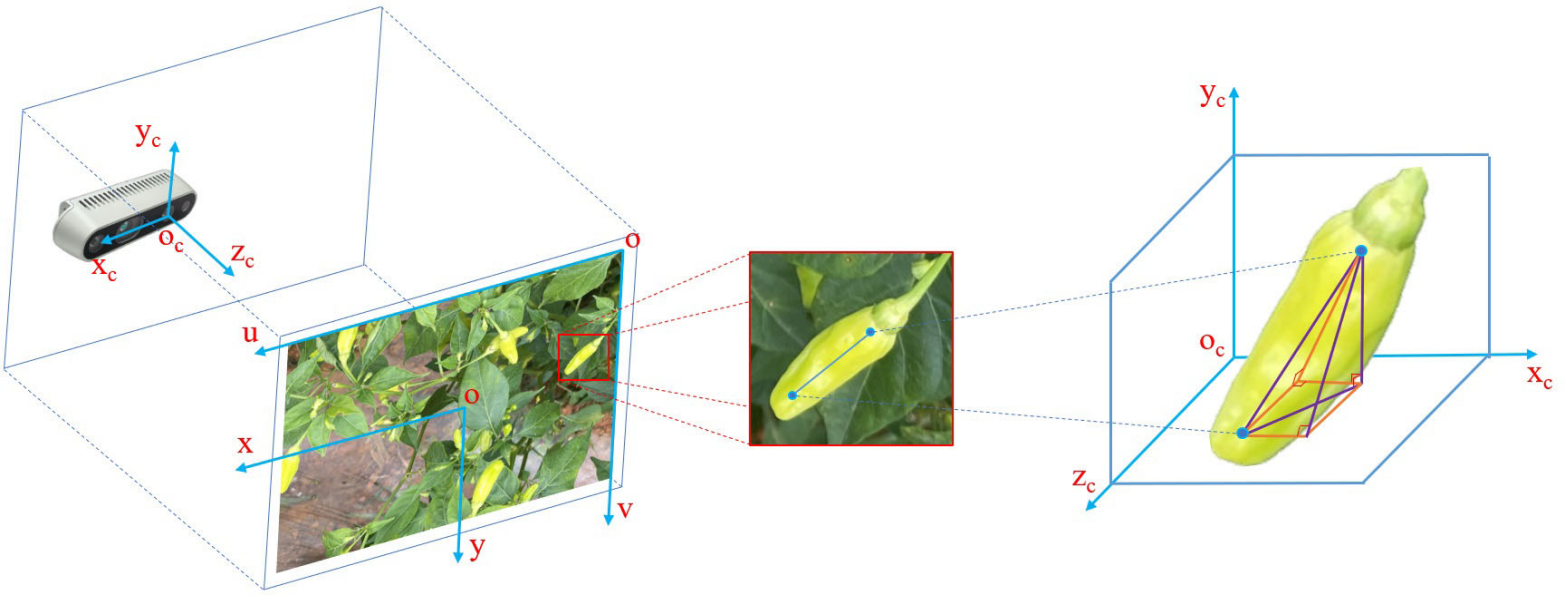
Schematic diagram of the coordinate systems.

The Xiaomila fruit fitting line is obtained based on a two-dimensional image, and its description method is based on the image pixel coordinate system. To obtain its posture in real space, the points in the pixel coordinate system need to be converted to the world coordinate system. The pixel coordinate system (o−uv) takes the upper left corner of the image as the origin of the coordinate system, and the unit is pixel; meanwhile, the image coordinate system (o−xy) takes the center point of the image as the origin of the coordinate system, and the unit is millimeter (mm); additionally, the camera coordinate system $\left(o_{c}-x_{c}y_{c}z_{c}\right)$ takes the optical center of the depth camera as the origin, and the unit is meter (m); moreover, the world coordinate system coincides with the camera coordinate system, as shown in **Figure 9**.

Before performing coordinate conversion, the Matlab-Camera Calibrator toolbox is utilized to calibrate the depth camera to obtain the camera’s internal parameter matrix and external parameter matrix. Then, the spatial point coordinates corresponding to the pixel point coordinates are calculated through Equation 5.


(5) $$Z_{c}\left[\begin{array}{c} u\\ v\\ 1 \end{array}\right]=KP\;\left[\begin{array}{c} \begin{array}{c} X_{w}\\ Y_{w}\\ Z_{w} \end{array}\\ 1 \end{array}\right]$$

where, $z_{c}$ represents the axial distance of the camera in the Z-axis, (u,v) is the pixel coordinate, K is the camera internal parameter matrix, P is the camera external parameter matrix, and $\left(X_{w},Y_{w},Z_{w}\right)$ is the point coordinate corresponding to the world coordinate system.

After the depth camera coordinate system is determined, a 3×1 translation matrix can be used to locate any point in the camera coordinate system. The conversion between the camera coordinate system and the Xiaomila coordinate system is represented by a 3×3 rotation matrix. Then, the position and attitude of the Xiaomila fruit can be determined by combining the translation matrix and rotation matrix. In this approach, the spatial position and spatial vector of the Xiaomila fruit are now known. Through inverse solution, the translation matrix and rotation matrix are obtained, thereby obtaining the rotation angle and translation distance of each joint. Finally, based on the rotation angle and translation information, the end effector is controlled to reach the designated position to complete the picking task. The translation matrix and rotation matrix are shown in Equations 6 and 7.


(6) PS=[pxpypz]

(7) RLS= (X^LS  Y^LS  Z^LS)= [r11  r12  r13r21  r22  r23r31  r32  r33]

where, PS is the translation matrix, RLS is the rotation matrix, S represents the depth camera coordinate system, and L represents the Xiaomila coordinate system; $p_{x}$ , $p_{y}$ , and $p_{z}$ are the center of gravity of the Xiaomila fruit relative to the camera, respectively. X^LS , Y^LS , and ${\;}^{S}{\hat{Z}}_{L}$ respectively represent the distance information of the Xiaomila coordinate system relative to the camera coordinate system along the x , y , and z  axes.

### 2.6 Evaluation metrics

#### 2.6.1 Evaluation of detection and segmentation

This paper takes detection precision (P), mean average precision (mAP), recall rate (R), F1 score, gigabit floating point operations per second (GFlops), and model weight file size as evaluation indicators. Precision is the ratio of the actual number of positives to the number of predicted positives. The higher the precision, the lower the false detection rate. The mean average precision is the mean of the average accuracy across all categories, and it is used to evaluate the accuracy of the entire model. Recall rate is used to evaluate the missed detection rate of the model. The F1 score measures the impact of precision and recall and is used to evaluate the stability of the model. GFlops represent the number of floating-point operations performed per second, and it is used to evaluate the computing performance of the model. The calculation formulas for these evaluation indicators are shown in Equations 8–11.


(8) $$P=\;\frac{TP}{TP+FP}$$

(9) $$mAP=\;\frac{1}{n}\sum_{i=1}^{n}AP_{i}$$

(10) $$R=\frac{TP}{TP+FN}$$

(11) $$F=\frac{2\times P\times R}{P+R}$$

where, TP represents the number of true positive values;  FP represents the number of false positive values; FN represents the number of false negative values; n represents the number of categories of identified objects, and $AP_{i}$ represents the average accuracy for category i .

#### 2.6.2 Evaluation of pose estimation

**Figure 10:**
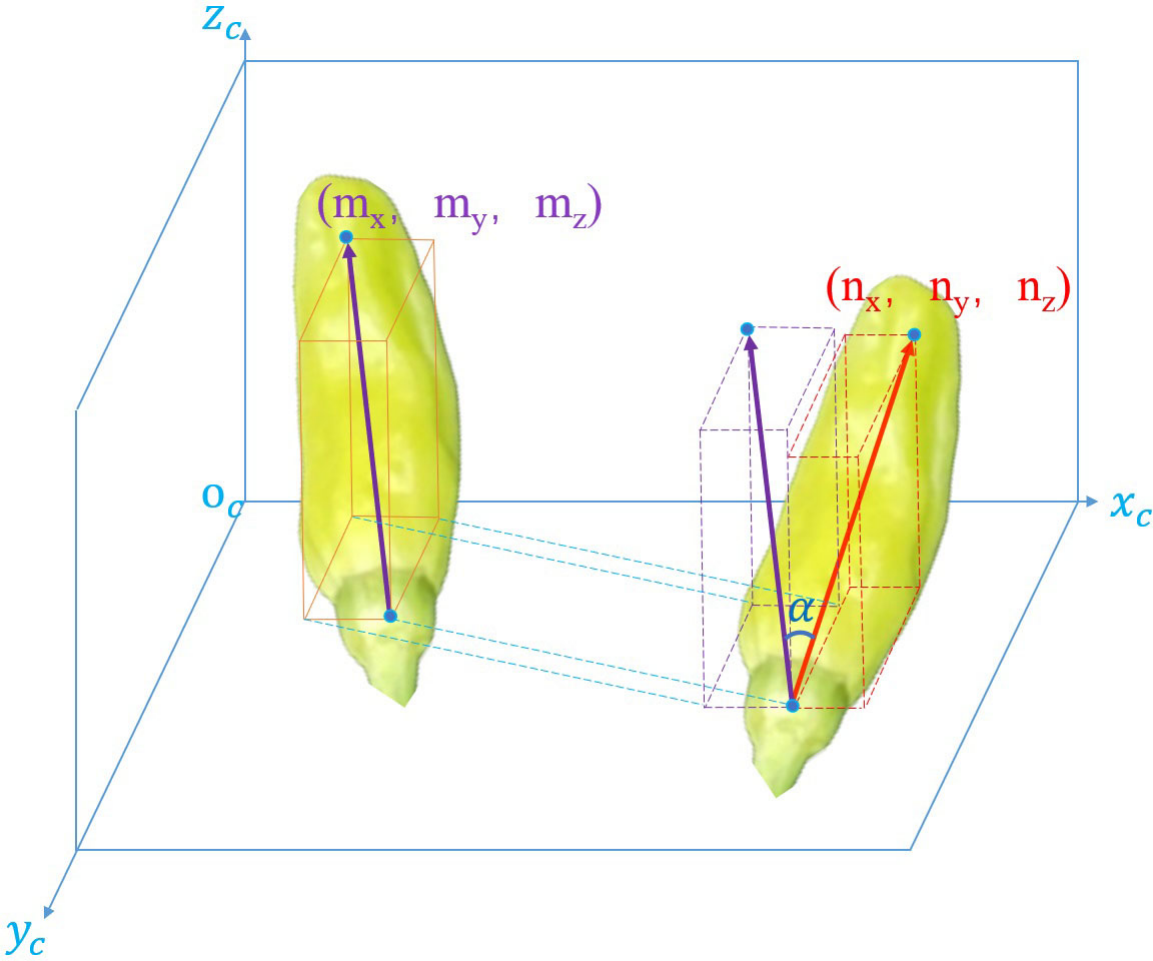
Diagram of error angle.


The error angle α is the angle between the actual space vector and the predicted space vector of the Xiaomila fruit. It is used to represent the error of the posture prediction algorithm, as shown in **Figure 10**. The calculation formula of α is shown in Equation 12:

(12) $$\alpha =\;arccos\frac{n_{x}m_{x}+n_{y}m_{y}+n_{z}m_{z}}{\sqrt{n_{x}^{2}+n_{y}^{2}+n_{z}^{2}}\times \sqrt{m_{x}^{2}+m_{y}^{2}+m_{z}^{2}}}$$

where, $m=\;\left(n_{x},n_{y},n_{z}\right)$ is the spatial vector of the Xiaomila fruit predicted by the attitude estimation algorithm, and $m=\;\left(m_{x},m_{y},m_{z}\right)$ is the actual spatial vector of the Xiaomila fruit. The smaller the error angle α, the closer the predicted posture is to the real situation.

### 2.7 Software

The hardware platform used for the experiment is a computer equipped with Intel Xeon W-2145 (16GB memory) and NVIDIA GeForce RTX2080Ti (11 GB video memory) and running 64-bit Windows 11 operating system. The Xiaomila target detection and segmentation model is trained using CUDA 11.6, Opencv, Pytorch framework, Python3.9 programming language, etc.

## 3 Results and discuss

### 3.1 Analysis of detection and segmentation

#### 3.1.1 Ablation experiment

To evaluate the impacts of the EMA attention mechanism and the DCNv3 convolution module on improving the detection performance of Xiaomila fruits, these two structures were introduced into the official YOLOv8 respectively. **Table 1** presents the impact of each module on the overall detection effect of the algorithm. The model performance was evaluated in terms of precision, recall, average precision, F1 score, floating point operations (FLOPs), and model weight size.

**Table 1:** Ablation experiments of different modules of PAE-YOLO.

Model	EMA	DCNv3	P (%)	R (%)	mAP (%)	F1 Score (%)	GFLOPs	Model Size/MB
YOLOv8n	×	×	86.5	78.8	87.5	82.5	8.1	6.2
	√	×	87.1	79.5	88.9	83.1	8.4	6.3
	×	√	87.3	78.1	87.6	82.4	7.4	5.7
	√	√	87.2	79.5	88.8	83.2	7.6	5.7

As shown in **Table 1**, several improvement strategies are effective in improving the model’s detection effect. Compared with the original YOLOv8n model, the recall rate and average precision of the model with the attention mechanism were increased by 0.7% and 1.4%, respectively. Meanwhile, the model weight was slightly increased, and the FLOPs reached 8.4G. After the convolution in the c2f module of the original model was replaced, the recall rate and average precision of the model were slightly improved compared to the original model, the model weight decreased by 8.1%, and the FLOPs dropped to 7.4G. Compared with the original YOLOv8n model, the average precision of the final PAE-YOLO model increased by 1.3%, the recall rate increased by 0.7%, GFLOPs decreased by 6.2%, the model size decreased by 8.1%, and the F1 score reached 83.2%.The results suggest that the EMA attention mechanism can improve the feature extraction capability of the model while adding a small number of parameters, and the DCNv3 convolution module enhances the portability and real-time detection performance of the model.

By combining the EMA attention mechanism and the DCNv3 deformable convolution network, PAE-YOLO not only improved the detection performance of Xiaomila fruits but also reduced the model’s calculation amount from 8.4G to 7.6G, and the model weight size dropped from 6.3M to 5.7M. Compared with the original YOLOv8n model, the FLOPs of PAE-YOLO were reduced by 6.2%, the model weight was reduced by 8.1%, the precision reached 87.2%, the recall rate reached 79.5%, the average precision reached 88.8%, and the F1 score was 83.2. Therefore, our method improves the algorithm performance in various indicators and reduces the algorithm’s computational complexity, which helps integrate the algorithm into picking robots for real-time applications.

#### 3.1.2 Comparative experiment

To verify the advantages of the model proposed in this paper in detecting Xiaomila targets, this study selected five classic detection models based on deep learning for performance comparison. **Table 2** shows the experimental results of Mobilenetv3, YOLOv5s, YOLOv7-tiny, YOLOv8n, and PAE-YOLO.

**Table 2:** Recognition results of different models on Xiaomila images.

Model	P (%)	R (%)	mAP (%)	F1 Score (%)	GFLOPs	Model Size/MB
Mobilenetv3	85.0	76.7	85.4	80.6	11.2	10.5
YOLOv5s	88.8	75.3	85.0	81.5	15.8	14.4
YOLOv7-tiny	85.7	82.8	89.5	84.2	13.0	12.3
YOLOv8n	86.5	78.8	87.5	82.5	8.1	6.2
PAE-YOLO	87.2	79.5	88.8	83.2	7.6	5.7

**Figure 11:**
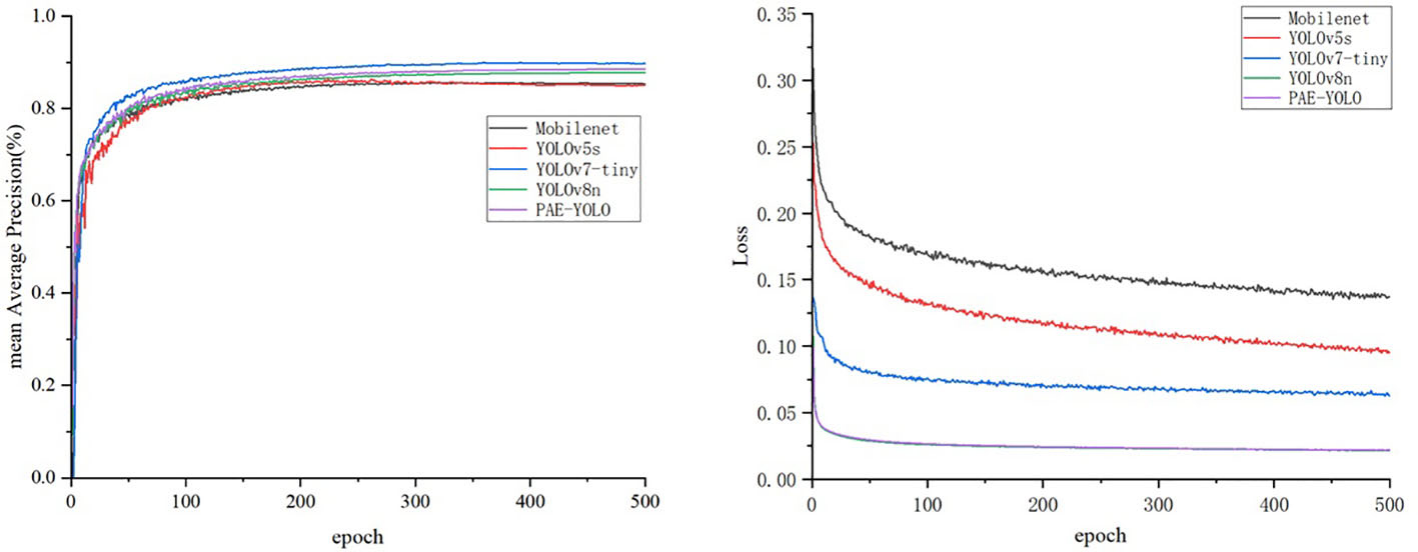
mAP and loss curves.

As illustrated in **Table 2** and **Figure 11**, compared with Mobilenetv3 and YOLOv5s networks, the recall rate of the PAE-YOLO model increased by 2.8% and 4.2% respectively, the mAP value increased by 3.4% and 3.8% respectively, and the model weight decreased by 45.7% and 60.4% respectively. Compared with the YOLOv7-tiny model, although the PAE-YOLO model had a slight decrease in precision and recall, the GFLOPs and weight decreased by 41.5% and 53.7%, respectively. The F1 score of PAE-YOLO ranked the best among the above-mentioned series of networks, with the smallest model weight and GFLOPs. Additionally, the PAE-YOLO model exhibited the lowest loss value and the fastest convergence speed during the training process.

**Figure 12:**
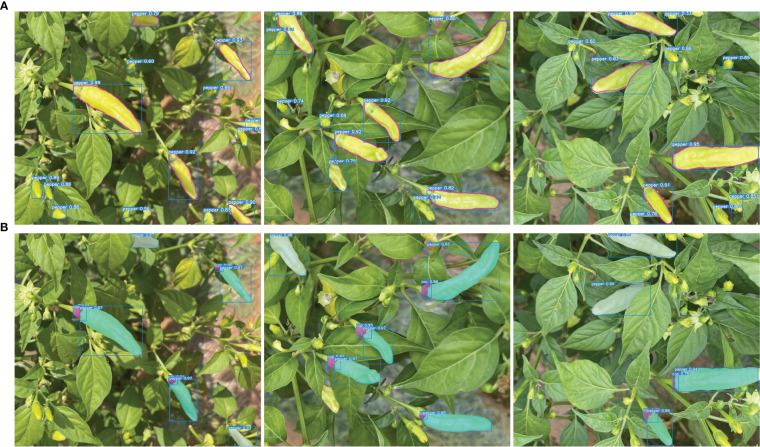
PAE-YOLO detection and segmentation results. **(A)** detection results of the xiaomila object, **(B)** segmentation results of the pickable xiaomila object.

These test results suggest that the PAE-YOLO network has a stronger overall performance in visual recognition of Xiaomila fruits. **Figure 12** shows the detection and segmentation results of the PAE-YOLO model. Specifically, (a) shows the detection results of the xiaomila object, in which the xiaomila with purple contour is the pickable object; (b) shows the segmentation results of the pickable xiaomila object.

### 3.2 Analysis of pose estimation effects

#### 3.2.1 Error angle analysis

In the actual farmland picking environment, if the error angle of Xiaomila’s attitude estimation falls within a certain range, the end effector of the picking equipment can achieve accurate picking. This study analyzes the error angles at different angles, as listed in **Table 3**.

**Table 3:** Error angle analysis.

Limit angle	Frequency	Average error	Standard deviation
Unlimited	1	18.63	13.89
<30°	0.844	13.75	4.94
<20°	0.711	11.98	2.91
<15°	0.556	10.63	1.44

**Figure 13:**
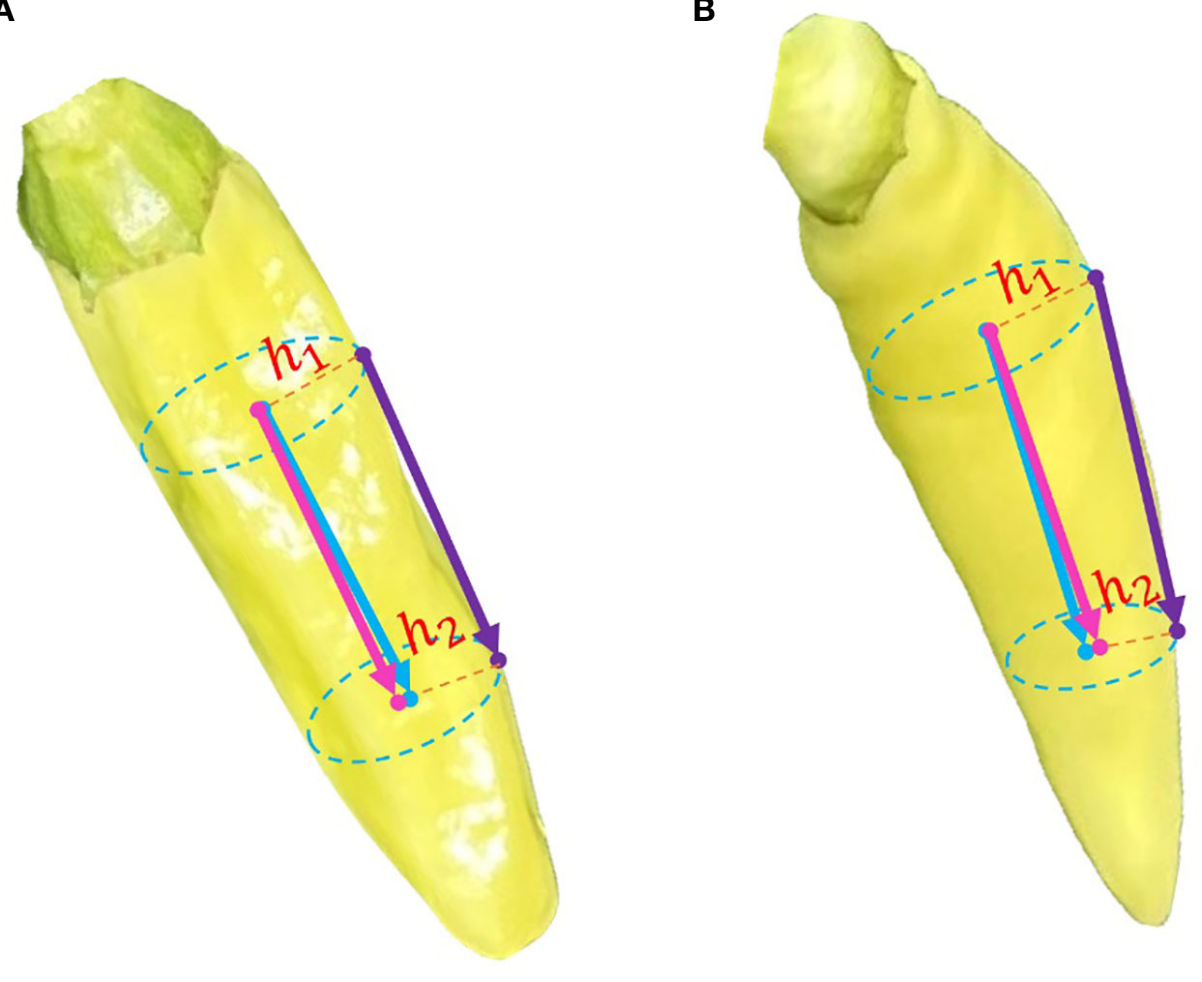
Example of spatial pose estimation of Xiaomila fruits. **(A)** spatial pose estimation of a Xiaomila fruit without bending, **(B)** spatial attitude estimation of a Xiaomila fruit in a curved state.

An example of the spatial pose estimated by the proposed pose estimation method is demonstrated in **Figure 13**. In this figure, the burgundy arrow represents the actual posture of the manually annotated pepper, the dark purple arrow represents the preliminary posture of the pepper estimated by the algorithm based on the surface points of the pepper, and the blue arrow represents the optimized posture of the pepper.

**Figure 13A** shows the spatial pose estimation of a Xiaomila fruit without bending, while **Figure 13B** shows the spatial attitude estimation of a Xiaomila fruit in a curved state. The posture of the Xiaomila fruit with a small curvature estimated based on surface points is basically consistent with the actual situation, while the estimation of the Xiaomila fruit with a large curvature based on surface points produces an error. This error may be ignored in complex farmland environments, resulting in an inability to correctly estimate the posture. This paper uses the two-dimensional Xiaomila fitting straight line as the symmetry axis to calculate the radial pixels at both end points of the estimated posture and then determines the inward offset distances h1 and h2 through the depth camera, thereby performing spatial analysis on the estimated posture.

#### 3.2.2 Analysis of different occlusion situations

**Figure 14:**
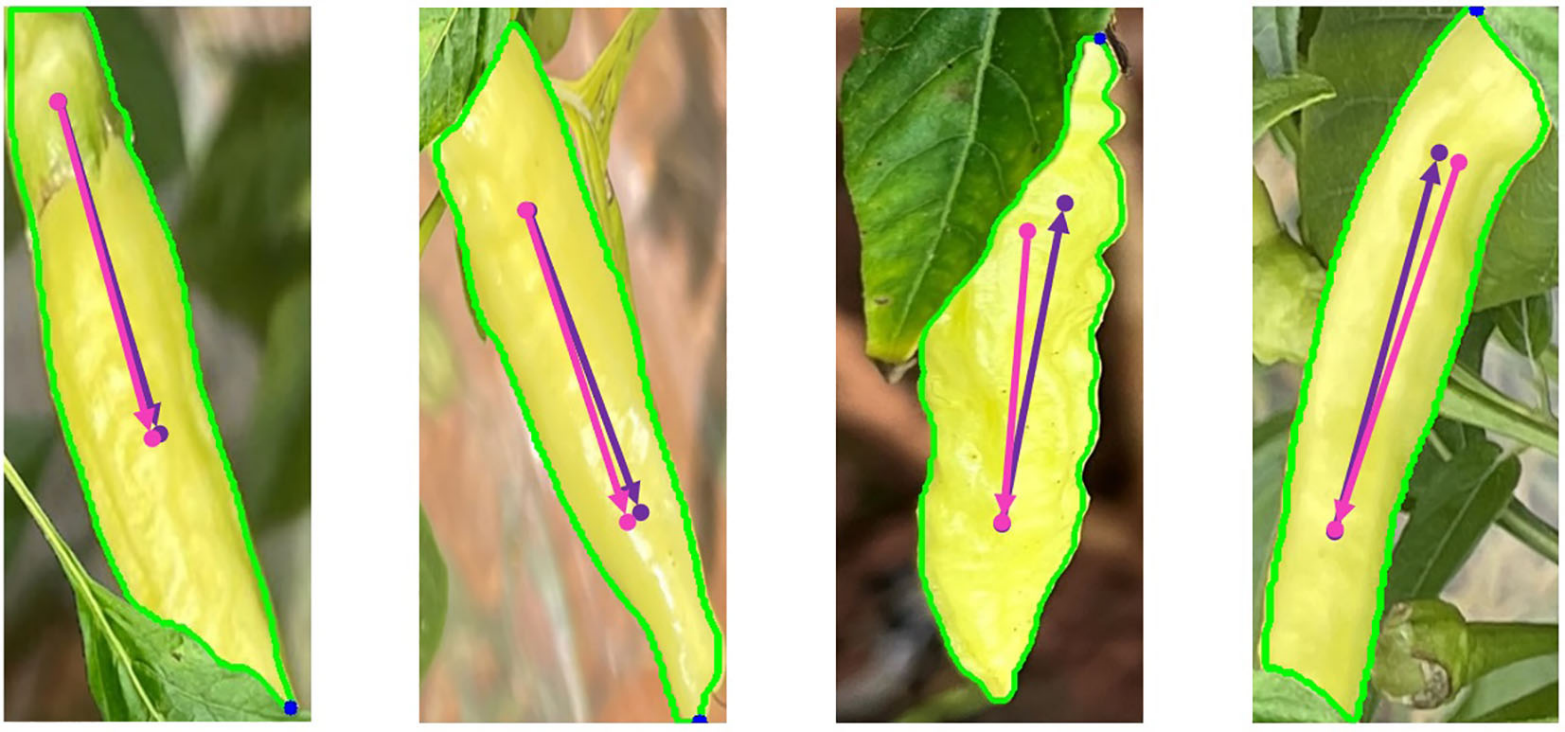
Classification of Xiaomila fruits occlusion. (1) Xiaomila pose estimation with the pepper cap not occluded, (2) (3) (4) Xiaomila pose estimation with the pepper cap occluded.

This paper discusses the pose estimation results under four different occlusion situations: the pepper cap is not occluded (a), the pepper cap is occluded but the occlusion does not produce a tip on the Xiaomila fruit (b), the pepper cap is occluded and the occlusion produces a tip on the Xiaomila fruit (c), and the pepper cap and tip are both occluded (d). In **Figure 14**, (1) shows the Xiaomila pose estimation with the pepper cap not occluded. (2)(3)(4) show the Xiaomila pose estimation with the pepper cap occluded. (3)(4) did not correctly determine the direction of the Xiaomila fruit. This is because (i) The pepper cap is occluded, and the tip angle formed by the occluded on the pepper cap part is smaller than the pepper tip angle. The attitude estimation algorithm makes an error when judging the orientation of the Xiaomila fruit. (ii) Both the pepper tip and pepper cap are occluded, and the algorithm cannot correctly identify and predict the specific orientation of the Xiaomila fruit.

**Figure 15:**
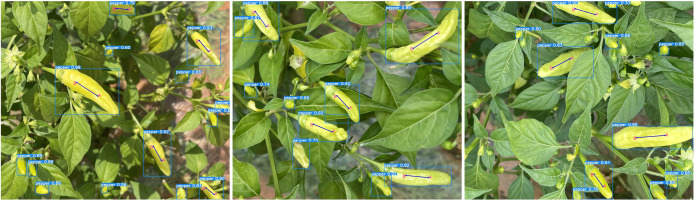
Attitude estimation renderings.

The attitude estimation error results under four different occlusion situations are presented in **Table 4**. The attitude estimation error when the pepper cap is not occluded is smaller than the attitude estimation error when the pepper cap is occluded. The average error angle is 23.19°. When the occluded cap is occluded, the algorithm fails to correctly identify the specific orientation of the pepper, thus affecting the attitude estimation effect. Since there are fewer situations (c) and (d) in practice, these two occlusion situations have less impact on the overall pose estimation effect. The final attitude estimation effect is shown in **Figure 15**, where end A represents the pepper tip, and end B represents the pepper cap.

**Table 4:** Pose estimation error under different occlusion situations.

Occlusion situation	Frequency	Average error	Standard deviation
a	0.667	15.68	5.87
b	0.196	16.69	5.63
c	0.059	160.97	6.37
d	0.078	122.31	55.11

## 4 Conclusion and future work

To solve the problems due to complex background, similar fruit color and background color, and different growth directions in the natural farmland environment, this paper constructed a Xiaomila target recognition data set, proposed an improved Xiaomila target detection model, and the spatial posture and occlusion of Xiaomila were analyzed. Specifically, the existing YOLOv8 target detection algorithm has been improved. The addition of the EMA attention mechanism can better capture the characteristic information of targets of different scales, and the deformable convolution module makes the model more lightweight. At the same time, the spatial position information of the pepper was exploited to describe the translation part of Xiaomila’s posture, and the transformation information of the fitted Xiaomila spatial vector relative to the depth camera coordinate system was utilized to describe the rotation part of Xiaomila’s posture. The advantage of this work is that no complex annotation model and calculations is required to obtain the expected estimation results, and can be better transplanted to embedded devices. In experiments, the mAP value of the improved PAE-YOLO model reached 88.8%, which was 1.3% higher than the original model. The model weight and GFLOPs were 7.6G and 5.7MB respectively, which are 6.2% and 8.1% lower than the original model, the loss value was the lowest during training, and the convergence speed was the fastest. Finally, an experimental analysis was conducted on Xiaomila’s posture and occlusion conditions. More than 85% of the cases where Xiaomila’s orientation was correctly estimated, with an average error angle of 15.91°. Under occlusion situations, 86.3% of the attitude estimation error angles were less than 40°, and the average error angle was 23.19°. Therefore, the improved detection model can accurately identify Xiaomila targets in complex environments, and can better estimate the target posture, and is suitable for visual picking of Xiaomila fruits.

However, current detection models still have some limitations. Some severely occluded Xiaomila targets cannot be correctly identified and estimated. For example, the pepper cap and the pepper peel are covered at the same time or the pepper cap is covered and the covering splits the pepper in two. Meanwhile, it remains to be seen whether the target recognition algorithm and attitude estimation method proposed in this article are applicable to other fruits. In future work, we will integrate the improved model into the robot motion control system to realize the automatic harvesting of Xiaomila in natural farmland environments.

## Data availability statement

The raw data supporting the conclusions of this article will be made available by the authors, without undue reservation.

## Author contributions

FW: Writing – review & editing. YT: Writing – original draft, Software, Validation, Writing – review & editing. ZG: Methodology, Writing – original draft. JJ: Data curation, Writing – original draft. YC: Resources, Writing – original draft. QX: Conceptualization, Writing – original draft. PH: Investigation, Writing – original draft. HZ: Writing – review & editing.
